# Transurethral drainage of prostatic abscess using pulsed Tm:YAG laser: a case report

**DOI:** 10.3325/cmj.2026.67.39

**Published:** 2026-02

**Authors:** Branimir Lodeta

**Affiliations:** Privatklinik Maria Hilf, Klagenfurt, Austria

## Abstract

Prostate abscesses are uncommon in a time of modern antibiotics. Nonetheless, surgical intervention and drainage are frequently required and may be performed via transperineal, transurethral, or transrectal approaches. Here, we report on a 78-year-old man who developed a prostatic abscess following joint replacement surgery. The patient was surgically treated with thulium laser enucleation of the prostate, performed using a pulsed solid-state thulium:YAG laser (Thulio®). He also received intravenous antibiotic therapy with meropenem. To our knowledge, this is the first report on transurethral drainage of a prostatic abscess using a pulsed Tm:YAG laser.

In men presenting with an acute urinary infection, a prostatic abscess should be considered if there is no response to initial antibiotic therapy or if the clinical condition worsens after an initial improvement (1,2). Prostatic abscess is most commonly comorbid with diabetes mellitus, while other predisposing factors include urinary tract outlet obstruction, prostate biopsy, long-term catheterization, prostatic manipulation, and immunosuppression (3,4).

Here, we report on a case of prostatic abscess due to *Pseudomonas aueruginosa* that was surgically drained using a thulium laser. There are no previous reports describing the use of a pulsed Tm:YAG laser for endoscopic (transurethral) drainage of prostatic abscesses.

## Case report

A 78-year-old patient had an indwelling urinary catheter inserted during total hip arthroplasty. Due to the development of gross hematuria, a urological consultation was sought. The urologist removed the catheter, whose balloon was inflated within the prostatic urethra, and subsequently placed a three-way catheter to facilitate continuous bladder irrigation and evacuation of intravesical coagulum.

After two days, the urine had cleared and the catheter was removed; however, the patient was unable to void spontaneously. Shortly thereafter, he developed chills and high-grade fever (Table 1). Laboratory evaluation indicated significant inflammation: C-reactive protein 213.0 mg/L (0-5 mg/L), procalcitonin 2.79 ng/mL (0-0.5 ng/mL), leukocytes 3.9 × 10^9^/L with 86% neutrophils, hemoglobin 8.4 g/dL (14-17 g/dL), platelets 62 × 10^9^/L (140-450 × 10^9^/L), and international normalized ratio 1.39 (0.84-1.24). The patient initially received empirical intravenous ampicillin/sulbactam. Blood and urine cultures both grew *Pseudomonas aeruginosa*, which was susceptible to meropenem. Therapy was therefore escalated to meropenem 1 g every 8 hours.

**Table 1:** Timeline of events and interventions*

Days of hospitalization	Event
Day 0 (September 15, 2025)	Total hip arthroplasty
	Catheter inserted with a balloon inflated in the prostatic urethra Gross hematuria, urologist consulted Three-way catheter with bladder irrigation placed
Day 2	Catheter removed
	Urinary retention Fever and chills IV ampicillin/sulbactam
Day 4	Cultures: *Pseudomonas aeruginosa*
	High CRP, low platelets Escalated to IV meropenem
Day 6	mpMRI Prostatic abscesses identified
	ThuLEP Laser enucleation and abscess drainage
Day 8 (postoperative day 2)	Patient afebrile
Day 11 (postoperative day 5)	Catheter removed No residual urine
Day 16 (postoperative day 10)	Recovery and discharge
	Afebrile Stable condition

*Abbreviations: ThuLEP – thulium laser enucleation of the prostate; IV – intravenous, CRP – C reactive protein.

**Figure 1:**
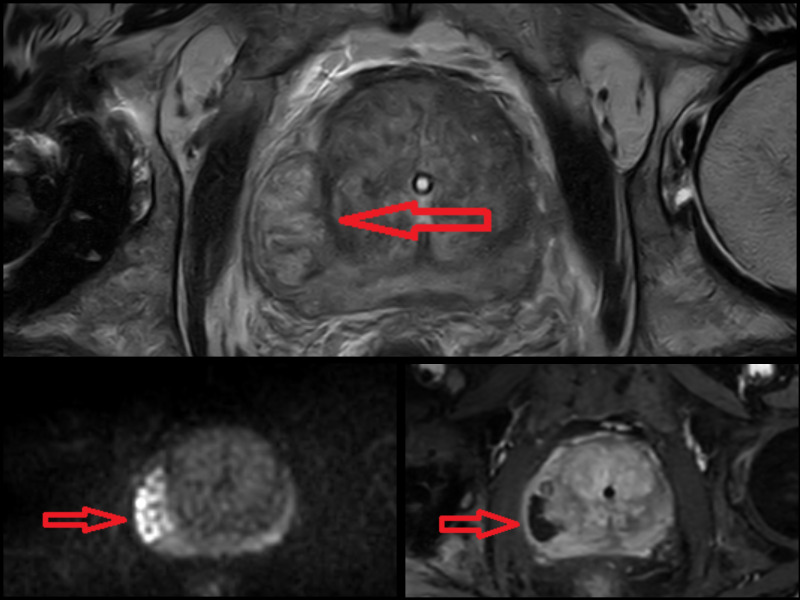
Magnetic resonance imaging of the prostate showing a prostatic abscess on T2-weighted imaging, diffusion-weighted imaging, and contrast-enhanced sequences (arrows).

**Figure 2:**
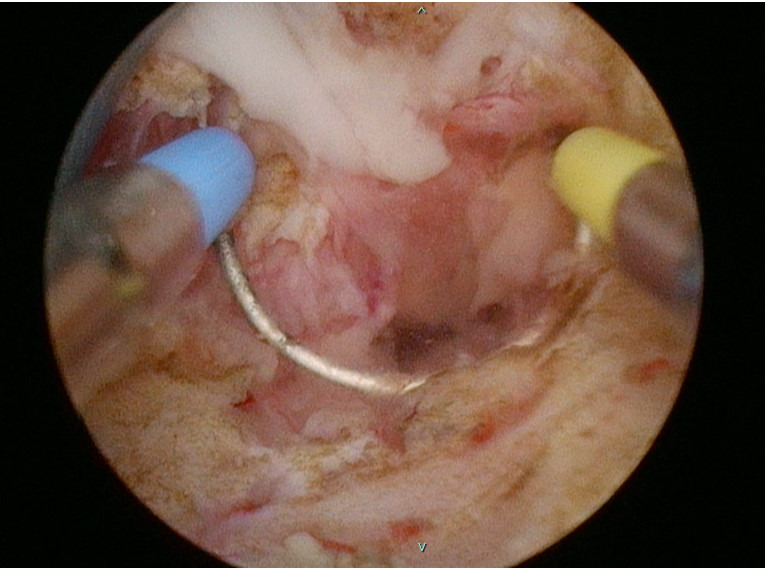
Intraoperative removal of dense purulent material from the prostatic abscess cavity using a bipolar resectoscope.

Given the absence of clinical improvement, multiparametic magnetic resonance imaging (mpMRI) was performed to exclude prostatic abscess. MpMRI demonstrated a markedly enlarged prostate gland (volume: 170 mL) containing two abscesses in the peripheral zone and multiple microabscesses in the transitional zone (Figure 1). On the same day, ThuLEP was used to deroof two peripheral-zone prostatic abscesses. A pulsed solid-state Tm:YAG laser (Thulio®, Dornier MedTech Systems GmbH, Wessling, Germany) was employed with a 600-μm fiber inserted via a 26-Fr continuous-flow resectoscope (Olympus, Tokyo, Japan). Laser power was 100 W (2 J, 50 Hz). Dense purulent material was subsequently mechanically evacuated from the abscess cavities using a bipolar transurethral resection electrode (Olympus) (Figure 2). The enucleation procedure lasted 42 minutes, followed by an additional 20 minutes for morcellation using the MultiCut Solo® system (JenaSurgical, Jena, Germany). Histopathologic examination of the evacuated tissue (112 g) confirmed benign prostatic tissue with evidence of inflammation.

The patient’s clinical status improved rapidly, with a marked reduction in inflammatory markers. He remained afebrile beginning on postoperative day two. The urinary catheter was removed on postoperative day five. Follow-up ultrasonography demonstrated complete bladder emptying without residual urine. The patient was discharged on postoperative day ten in a stable condition.

## Discussion

The risk of developing a prostatic abscess is increased in individuals with diabetes mellitus, HIV infection, prostatitis, neurogenic bladder, voiding dysfunction due to bladder outlet obstruction, indwelling catheters, or a history of urinary tract instrumentation (5). The leading cause of acute bacterial prostatitis and subsequent prostatic abscess is *Escherichia coli*, while other common pathogens are *Pseudomonas aeruginosa*, *Klebsiella species*, and *Enterococcus* (6). If not properly treated, a prostatic abscess may progress to urosepsis and become life-threatening (7). Traditionally, transrectal ultrasound has been used to evaluate prostatic abscesses due to its high sensitivity, low cost, and usefulness in guiding therapy (1). Computed tomography (CT) is a widely available and clinically useful imaging modality and should be employed when TRUS does not provide definitive findings or when spread beyond the prostate is suspected. In such cases, CT typically shows a swollen prostate containing nonenhancing fluid-density areas, which may exhibit internal septations or a surrounding rim enhancement (8,9). In this case, CT imaging was considered less appropriate due to expected artifacts from the hip prosthesis, which could compromise image quality in the pelvic region. Additionally, contrast administration was avoided since the patient had undergone nephrectomy several years earlier. Owing to its exceptional soft-tissue resolution, MRI serves as a valuable modality for delineating the extent of a prostatic abscess and identifying extraprostatic extension (10). Characteristic findings on MRI include a hyperintense centrally necrotic region on T2-weighted and high b-value diffusion-weighted sequences with a corresponding low apparent diffusion coefficient, while the peripheral zone shows rim enhancement.

In the present case, the two principal predisposing factors were benign prostatic hyperplasia with bladder outlet obstruction and an improperly positioned indwelling catheter.

Conservative management with intravenous antibiotics alone may be appropriate for small, unifocal abscesses (<1 cm) (11). Surgical interventions for a prostatic abscess are transrectal ultrasound-guided aspiration, transperineal drainage, transurethral deroofing or resection of the abscess cavity, and, in selected cases, enucleation of the prostate using holmium laser (12).

Patients treated with needle aspiration tend to have prolonged hospitalization and a higher incidence of recurrence; moreover, this approach is generally unsuitable for multiloculated abscesses or in cases complicated by infravesical obstruction secondary to prostatic enlargement (13).

Holmium laser technology has been previously used for the treatment of a prostatic abscess, offering several advantages, including the ability to completely enucleate the infected prostatic adenomatous tissue (12,14). Endoscopic laser enucleation of the prostate using a pulsed solid-state thulium:YAG laser is a safe, recognized, and efficacious technique for the management of prostatic enlargement (15).

This case highlights an alternative therapeutic strategy for managing a prostatic abscess through the application of advanced laser technology. We performed endoscopic enucleation of adenomatous tissue containing microabscesses within the transitional zone and deroofing of peripheral-zone abscesses using the novel Thulio® laser. In this way, we treated both the prostatic abscess and the underlying benign prostatic hyperplasia responsible for bladder outlet obstruction and residual urine.

The present case study suggests that pulsed solid-state Tm:YAG laser may represent a promising option for abscess drainage by providing precise treatment and effective hemostatic control. However, given the inherent limitations of a case report, these findings cannot be generalized. Further studies with larger patient cohorts are needed before this technique can be recommended for routine use in everyday clinical practice.
